# SARS-CoV-2 infection in a patient with propionic acidemia

**DOI:** 10.1186/s13023-020-01563-w

**Published:** 2020-10-28

**Authors:** Anna Caciotti, Elena Procopio, Francesca Pochiero, Silvia Falliano, Giuseppe Indolfi, Maria Alice Donati, Lorenzo Ferri, Renzo Guerrini, Amelia Morrone

**Affiliations:** 1 Molecular and Cell Biology Laboratory, Paediatric Neurology Unit and Laboratories, Neuroscience Department, A. Meyer Children’s Hospital, Viale Pieraccini n. 24, 50139 Florence, Italy; 2 Metabolic and Muscular Unit, A. Meyer Children’s Hospital, Florence, Italy; 3 Paediatric and Liver Unit, Meyer Children’s University Hospital, Florence, Italy; 4 Department of Neurosciences, Psychology, Pharmacology and Child Health, University of Florence, Florence, Italy

**Keywords:** Propionic academia, SARS-CoV-2, COVID-19, *PCCB*

## Abstract

We describe a 14-month-old boy, with a previous diagnosis of propionic acidemia (PA) by expanded newborn screening, who, admitted for a suspected metabolic crisis, tested positive for SARS-CoV-2. Since propionic acidemia was diagnosed, the patient has followed the recommended diet for this inborn error of metabolism. Although propionic acidemia patients are at a high risk of suffering metabolic crises, frequently associated with permanent clinical complications, psychomotor development of this patient was normal. The SARS-CoV-2 infection (at about 1 year of age) caused the patient’s first metabolic crisis. However, his clinical course was in keeping with a mild clinical form of COVID-19, and he recovered without experiencing severe clinical consequences. We describe this patient in order to improve the knowledge about follow up of PA patients identified by newborn screening and to increase the limited number of reports of SARS-CoV-2 infection in children with comorbidities, especially inborn errors of metabolism.

## Background

Propionic acidemia (PA) (MIM #606054) is a multisystemic inborn error of the catabolic pathway of branched-chain amino acids (isoleucine, valine, methionine and threonine). It is caused by mutations in the *PCCA* and *PCCB* genes, encoding alpha and beta subunits (UniProtKB—P05165 and P05166) of the mitochondrial enzyme propionyl-CoA carboxylase (PCC, EC 6.4.1.3) [1]. Biochemical characteristics include metabolic acidosis, ketosis, hyperammonaemia, altered glycemia, neutropenia, anemia and thrombocytopenia [1].

PA is characterised by high levels of 3-hydroxypropionate, methylcitrate, tiglylglycine and propionylglycine in urine [2]. In recent years, an increasing number of patients have been detected by newborn screening (NBS) programs which check for elevated levels of C3 (propionyl carnitine) in dried blood spots (DBS) taken from 48 to 72 h after birth [3].

The altered catabolism of proteins in PA causes severe psychomotor impairment, seizures, movement disorders, gastrointestinal symptoms, cardiomyopathy, renal involvement, hematological abnormalities, osteoporosis, immune dysfunctions and other symptoms. Most affected patients present the severe neonatal form, although later onset and milder forms are described [4].

An acute metabolic decompensation, which can result in selective organ damage, especially brain injury, can be avoided with therapy [1] and controlled diet [5]. In managing PA patients, the key objective is to prevent acute episodes [5]. Acute decompensations, whose initial management is critical, can be triggered by fever, vomiting, prolonged fasting, gastroenteritis and other infections [5, 6]. The health gains of NBS for PA in overall outcome are modest, even if mortality in patients detected by NBS is lower than in the group detected by selected metabolic screening [3, 5–7].

The recent epidemic of the 2019 novel coronavirus SARS-CoV-2, has caused significant morbidity and mortality worldwide. In general, children appear to have a milder clinical course compared to adults [8–11]. Little is known about SARS-CoV-2 infection in children with comorbidities (such as congenital heart, lung and airway diseases, chronic heart and kidney diseases, malnutrition, tumors, diabetes, immunodeficiency or hypoimmunity) and little information is available on the effects of the infection in pediatric patients with congenital inborn errors of metabolism (IEM) [8, 12]. However, emerging guidelines have been proposed to manage eventual SARS-Co V2 infection in lysosomal diseases and on inherited heart diseases [13–15]. In addition, it has been reported that a patient affected by mucolipidosis type II died because of pneumonia complicated by acute respiratory distress syndrome (ARDS) [16].

Here we describe a 14-month-old patient followed by our team since PA was detected by NBS and confirmed by *PCCA* and *PCCB* gene sequencing, who was recently infected by SARS-CoV-2.

## Case report

At birth all the patient’s growth and vital parameters were normal and his APGAR score was 9^1^–10^5^. PA was diagnosed by NBS 5 days after birth. Molecular analysis of the PA genes (*PCCA* and *PCCB*), performed by next generation sequencing procedures (Nextera Flex technology, Illumina), identified compound heterozygosity for two previously reported mutations in the *PCCB* gene, the NM_000532: c.337C > T p.(Arg113*) [17] and the c.1298dupA p.(Ala434Glyfs*7) [18]. Both mutations, each of which was present at the heterozygous status in one of the parents, cause premature stop codons, which are likely to prevent production of normal and functional PCCB proteins.

On his fifth day of life, due to hyperammoniemia (357 μmol/L, n.v < 50) and metabolic acidosis, the child was admitted to our hospital. Protein intake was stopped and intravenous glucose and lipids, l-carnitine, *N*-Carbamylglutamate, sodium benzoate and arginine hydrochloride were administered (Table 1). 24 h after hospitalization, ammonemia levels were normal and natural proteins (human milk) were reintroduced. Therapy with ammonia scavengers was suspended on the 9th day after birth (Table 1). Transfontanellar ultrasound, cardiologic evaluation, hearing and ophthalmological screening were all normal.

**Table 1:** Patient’s follow-up

	Before Sars-Cov-2 infection	During Sars-CoV-2 infection	After Sars-Cov-2 infection
Therapy	l-Carnitine 750 mg × 3/die *N*-carbamylglutamate (Carbaglu 80 mg/kg/die) Metronidazolo 125 mg in 2 doses for 15 days per month Dycoflor 5 drops/die Vitauno (DMF) 10 drops/die	l-Carnitine 750 mg × 3/die *N*-carbamylglutamate (Carbaglu 100 mg/kg/die) Dycoflor 5 drops/die Vitauno (DMF) 10 drops/die	l-Carnitine 750 mg × 3/die *N*-carbamylglutamate (Carbaglu 80 mg/kg/die) Metronidazolo 125 mg in 2 doses for 15 days per month Dycoflor 5 drops/die Vitauno (DMF) 10 drops/die
Diet	Milk (Nidina 1, Nestlè, 160 ml × 4/die) Glycolipid and Vitamin supplement (BasicP, Milupa, Nutricia, 20 ml × 4/die) 2 salt meals/day (protein intake 13.5 g/die	Protein intake was stopped for 24 h. During this 24 h: -Glucose-electrolyte infusion with a glucose intake of 7 mg/Kg/min -Fat emulsion infusion (2 g/kg/day, 10% medium-chain triglyceride + 10% long-chain triglyceride)	Milk (Nidina 1, Nestlè, 160 ml × 4/die) Glycolipid and Vitamin supplement (BasicP, Milupa, Nutricia, 20 ml × 4/die) 2 salt meals/day (protein intake 13.5 g/die)

During the first year of life, as serum ammonia levels continued to be slightly above normal values, carnitine dosage was progressively increased and therapy with *N*-carbamylglutamate and metronidazole was introduced at 11 months of age. Protein intake has always been kept within safe limits. Clinical and biochemical parameters remained satisfactory during the clinical course. The patient’s neurodevelopmental milestones remained normal. He gained head control at 3 months of age, began to sit without support at 6 months and started to say some words at 12 months.

At the last follow-up appointment, therapy included l-Carnitine, *N*-carbamylglutamate (80 mg/kg), Vitamin complex [Vitamin C, E, A, D3, B12 and folic acid], 12.5 mg/kg Metronidazole in 2 doses for 15 days per month (Table 1).

**Fig. 1:**
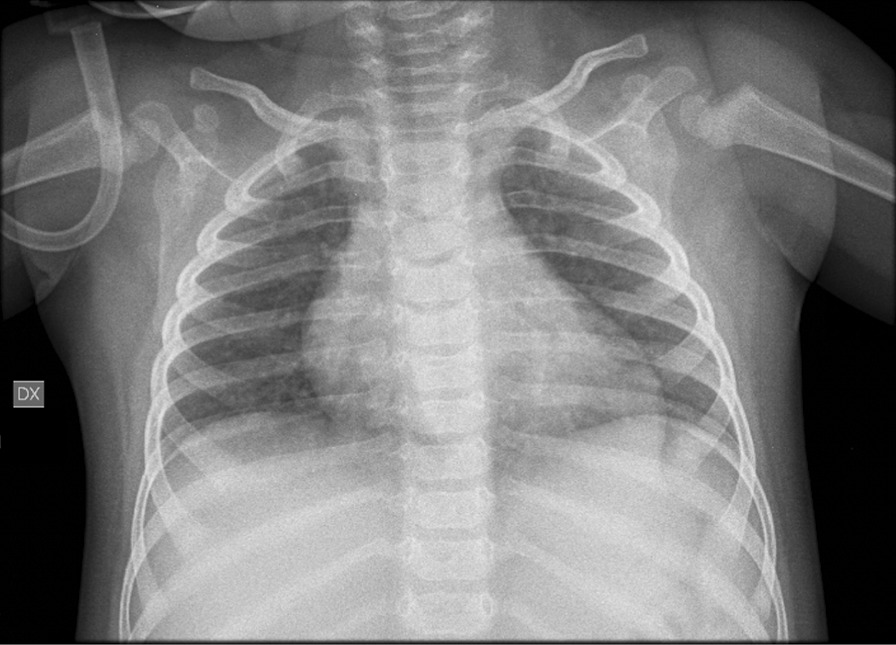
Patient’s chest X-ray A-P projection. Widespread mild accentuation of the broncho-vascular texture with blurred aspect

At 1 year of age the patient was admitted to our hospital due to a combination of symptoms including vomiting, drowsiness, cold sweating, skin pallor, and dyspnea. His parents referred a low-grade fever in the previous days. A metabolic crisis was suspected. He was given O_2_ therapy due to oxygen saturation values (as measured by pulse oximetry) being below 90% (Table 2). Dyspnea resolved in 2 h with O_2_ therapy and the child remained in relatively good health and was able to self-feed during the crisis. Chest- Brain CT and ECG were normal; blood testing revealed hyperammonemia and hyperglycemia (see Table 2). Chest echography showed a widespread increase of B lines with small subpleural thickenings. Glucose infusion and 100 mg/kg *N*-carbamylglutamate administration was followed by rapid recovery with no subsequent metabolic crises. Three days after admission fever and diarrhea appeared. Due to the occurrence of high fever (38.5 °C), SARS-CoV-2 infection was suspected and diagnosed by nasopharyngeal swab. Chest X-ray showed widespread, albeit mild accentuation of the broncho-vascular texture with a blurred aspect (Fig. 1). The patient’s biochemical parameters observed during hospitalization and Sars CoV-2 infection are presented in Table 2. The child was discharged 2 days after his fever resolved, with no further treatment. Both parents were infected by SARS-CoV-2 but remained asymptomatic. Currently, the patient has normal psychomotor development and his family complies with therapeutic recommendations. He is now 16 months of age and has started walking (at 15 months of age).

**Table 2:** Biochemical data of PA patient during Sars-CoV-2 infection

Day of the crisis	1				2	3	5	7
	10:00 a.m	11:00 a.m	2.00 p.m	5:00 p.m				
*Vital parameters*								
Sat0_2_ (%)								
95–100	90		81.1*		92.7			
*ABG parameters*								
PH (–)								
N.V. 7.35–7.45	7.31	7.24*	7.35		*7.42*			
PCO_2_ (mmHg)								
N.V.80–100	54*	71*	44.5*		34.0*			
HCO3- (mmol/L)								
N.V.21–30	23.8	30.4	22.7		23.1			
cLac (mg/dl)								
N.V. 5–14			21*		16*			
*Clinical chemistry*								
NH_4_ (µmol/L)								
N.V. < 50	145*		113*	113*	49	42		67*
Glycemia (mg/dl)								
N.V. 60–100	162*	211*	134*	134*	107*	101*	118*	126^*^
Creatinine (mg/dl)								
N.V.0,10–0,40			0.16		0.18	0.21	0.22	
*Hematology*								
WBC (× 10^3/µl)								
N.V.3.50–14.00			5.65		6.83	8.20	4.52	10.87
PLT (× 10^3/µl)								
N.V. 210–590			145*		133*	144*	188*	299
Lymphocytes (%)								
N.V. 30–70			37.3		78.4*	44.7	49.2	56

^*^Out of range values are italicised; *n.v.* normal values, *ABG* arterial blood gas, An infection of the urinary tract was excluded

## Discussion

SARS-CoV-2 is a single stranded RNA virus that can cause a severe respiratory syndrome [19]. Fever and cough are the most common symptoms; additional non-specific symptoms include dyspnea, myalgia or fatigue and lymphopenia, while vomiting and diarrhea are less commonly observed [8].

Systemic reviews of COVID-19 in pediatric patients suggests that the clinical course of SARS CoV-2 infection in children is milder than in adults [12, 19, 20]. However, the impact of this infection on the clinical status of children with an inborn metabolic disorder is not well known [16, 19].

The child we here describe, who is affected by a severe form of PA, was the first patient affected with SARS-CoV-2 infection in our pediatric hospital. The infection was diagnosed at an early stage of the pandemic outburst in Italy. At that time, routine safety protocols had not yet been established in Italian hospitals or in the community. The patient was tested for SARS-CoV-2 because he exhibited dyspnea and fever, which we knew to be suggestive of COVID-19. Diagnostic investigations did not reveal any major abnormality at presentation or during the course of the infection, although mild involvement of the lung texture was detected by echography and by chest X-ray.

Current national and regional ordinances require patients admitted to hospital to be tested for SARS-CoV-2 using both nasopharyngeal swabs and serological testing (Screen Test COVID-19, 2019-NCOV IGG/IGM rapid test cassette, Screen Italia srl). Since this patient tested positive for SARS-CoV-2, our hospital has registered 12 additional instances of metabolic decompensation in pediatric patients affected with inborn errors of metabolism: 6 methylmalonic acidemias, 4 PAs, 1 argininosuccinate lyase deficiency and 1 glycogenosis. COVID-19 was excluded in all of them and in their parents.

Fever is one of the most frequent causes of metabolic decompensation in organic acidurias [5]. Adherence to l-carnitine and *N*-carbamylglutamate treatment and dietary restrictions can prevent decompensation/crisis improving survival rate even in patients with the severe form of PA [4]. Appropriate management of acute metabolic crises is crucial for normal intellectual development in PA patients [4]. Recent literature confirms that hyperammonemia and metabolic decompensation have a great impact on patients’ outcome, and that even when diagnosis is a result of NBS, outcomes are not necessarily better [7, 21, 22].

Long-term complications affecting various organ systems are common in PA patients, and impaired physical development including growth retardation and psychosocial development may further complicate the outcome for young/adult PA patients [4, 22]. There is still controversy about the optimum natural protein intake for patients with PA and different therapeutic regimens are used by different metabolic centers [4]. During SARS-CoV-2 infection of the here reported patient, hyperammonemia and glycemia promptly normalised and the patient was discharged a few days later, after his fever resolved. COVID-19 did not prompt severe metabolic decompensation in this patient and the used treatments prevented short-term complications. We suggest that for this patient, the protocols used to treat hyperammonemia at 6 days of age, strict dietary management, ongoing therapy and prompt intervention on the patient’s recent admission may have helped lessen the impact of the metabolic decompensation.

The continuous reassessment of COVID-19 care plans is crucial during the current pandemic. Sharing observations about the evolution and management of COVID-19 infection in pediatric patients with underlying inborn errors of metabolism through rapid publication is vital [23].

## Data Availability

Not applicable.

## Abbreviations

PA: Propionic academia
NBS: Newborn screening
